# Novel Application of ^32^P Brachytherapy: Treatment of Angiolymphoid Hyperplasia with Eosinophilia in the Right Auricle with 8-Year Follow-Up

**DOI:** 10.1089/cbr.2018.2468

**Published:** 2018-09-01

**Authors:** Jinshan Zhang, Yuan Li, Ge Wen, Yongmei Deng, Hongxia Yao

**Affiliations:** Department of Radiation Oncology and Nuclear Medicine, The Third Affiliated Hospital of Guangzhou Medical University, Guangzhou, P.R. China.

**Keywords:** angiolymphoid hyperplasia with eosinophilia (ALHE), brachytherapy, phosphorus-32

## Abstract

***Background:*** Angiolymphoid hyperplasia with eosinophilia (ALHE) is a distinctive benign vascular disease that can be challenging to treat due to inconsistent results for various treatment modalities such as surgical excision, corticosteroids, radiotherapy, laser therapy, and other therapies, so novel approaches are needed to improve treatment outcomes.

***Materials and Methods:*** ALHE on the right auricle of a 54-year-old Chinese woman underwent brachytherapy using ^32^P simple drug membranes for five times. The ^32^P brachytherapy involving simple drug membranes of brachytherapy began by diluting a ^32^P solution with 0.9% NaCl solution to produce a radioactivity of 69.2–74.7 MBq/mL(1.87–2.02 mCi/mL). The drug membranes were removed between 48 and 72h after application. There were intervals ranging from 65 to 72d between the membrane application periods, and the last treatment was in June 2010.

***Results:*** After the ^32^P brachytherapy, follow-up results over the course of 8 years were promising. The regional symptoms disappeared, the right preauricular swelling decreased, the subcutaneous nodules decreased in size, the exudate disappeared, and the skin appearance improved.

***Conclusions:*** This case indicated that ^32^P brachytherapy may represent a novel ALHE treatment method that produces a favorable long-term outcome.

## Introduction

Angiolymphoid hyperplasia with eosinophilia (ALHE), first described in 1969 by Wells and Whimster,^1^ is a rare benign vascular disease. Despite its benign nature, ALHE presents a therapeutic dilemma,^2^ as persistent or recurrent lesions with clear symptoms may require treatment. Various modalities such as surgical excision, corticosteroids, radiotherapy, laser therapy, and other therapies have been used to treat ALHE. Surgical excision appears to be the most effective treatment for ALHE, but clinicians should anticipate an ∼40% recurrence rate; so novel approaches are needed to improve treatment outcomes for this condition.^3^ In this study, the authors report a case of ALHE occurring on the right auricle that had improved presentation for over 8 years after ^32^P brachytherapy.

## Case Presentation

**FIG. 1.**
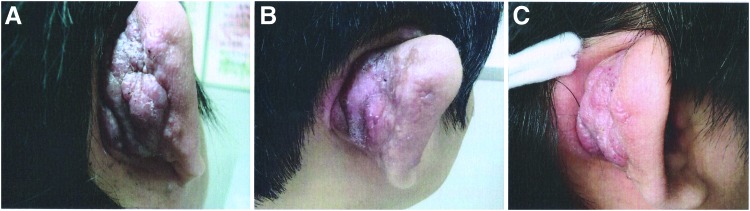
Multiple nodules under the subcutaneous tissues on the right auricle **(A)**. Three- **(B)** and eight-year **(C)** follow-up of ALHE on the right auricle treated with ^32^P brachytherapy. ALHE, angiolymphoid hyperplasia with eosinophilia. Color images available online at www.liebertpub.com/cbr

A 54-year-old Chinese woman with a 5-year history of multiple nodules under the subcutaneous tissues on the right auricle (Fig. 1A) was referred to their hospital in April 2009. The patient also suffered from itching and occasional tingling in the right auricle. Topical corticosteroids and oral antihistamines were prescribed, but the condition repeatedly recurred. On examination, erythematous or violaceous papules and nodules were present in the right dermis and subcutaneous tissues, and auricle swelling was observed. No regional lymphadenopathy or other pathological findings were evident.

**FIG. 2.**
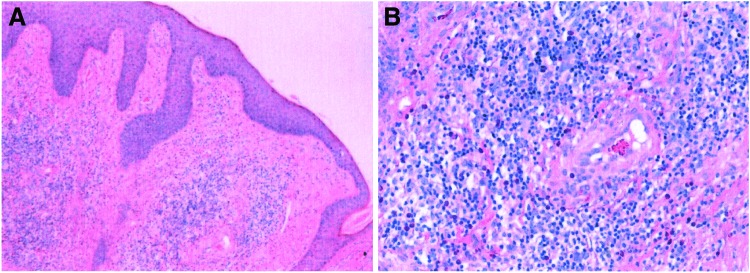
Small blood vessels proliferated in the dermis and were accompanied by infiltrating eosinophils and lymphocytes, epidermal hyperplasia with hyperkeratosis, and epithelial foot extensions. Hematoxylin and eosin staining at **(A)** low (40 × ) and **(B)** high power (200 × ) magnification. Color images available online at www.liebertpub.com/cbr

Laboratory data, including eosinophil count and total serum immunoglobulin (Ig)E, were within normal limits. A biopsy was performed on the lesion, and the pathological diagnosis was ALHE (Fig. 2).

After the patient presented to their department, brachytherapy with ^32^P simple-drug membranes was performed on the lesions. The patient underwent ^32^P brachytherapy treatment five times. The ^32^P brachytherapy involving simple-drug membranes of brachytherapy began by diluting a ^32^P solution (Beijing Atoms High-Tech Ltd. Co.) with 0.9% NaCl solution to produce a radioactivity of 69.2–74.7 MBq/mL (1.87–2.02 mCi/mL). The lesion area was covered with transparent plastic film and cellulose qualitative filter paper (Grade 1) as a medicine film. The size of the ^32^P simple-drug membranes was determined using carbon paper, and the membranes were prepared by evenly applying the diluted ^32^P to filter paper, which was then allowed to dry. Electric soldering was used to close the transparent plastic film. The treatment area was disinfected using iodine tincture, and the prepared ^32^P simple-drug membranes were pressed tightly to the lesion. The drug membranes were removed between 48 and 72 h after application, and the membranes were properly disposed of as radioactive waste. There were intervals ranging from 65 to 72 d between the membrane application periods, and the last treatment was in June 2010. After the ^32^P brachytherapy, follow-up results over the course of 8 years were promising. The regional symptoms disappeared, the right preauricular swelling decreased, the subcutaneous nodules decreased in size, the exudate disappeared, and the skin appearance improved (Fig. 1B, C).

## Discussion

ALHE, also called epithelioid hemangioma, is a rare benign proliferative disease. The most common locations for ALHE are the ear and periauricular area (36.3%).^3^ The lesions typically present as well-circumscribed papules or nodules that are red, brown, or purple in color,^4^ but the diagnosis should be histologically confirmed and not confused with Kimura's disease.^4–6^ More than half of ALHE patients (53.4%) present with a single lesion, and the remainder have multiple lesions. Only 15.4% of ALHE cases are asymptomatic, whereas the majority of ALHE patients suffer from at least one symptom, including pruritus (36.8%), bleeding (25.3%), or pain (20.2%).^3^ Furthermore, some lesions can become infected,^7^ pulsatile, or ulcerated. Thus, timely and effective treatment of ALHE is necessary for symptomatic relief, to address cosmetic concerns and to prevent tissue deterioration.

Many approaches have been used to treat ALHE, including surgical excision,^2,3,5,8–10^ steroids, and other drugs,^11–16^ as well as biological and physical therapy.^17–22^ Surgery is the mainstay of ALHE treatment, although surgery is less successful for cases with poorly delineated multiple nodules. Moreover, surgery can be disfiguring and technically difficult, especially in the periauricular region. Other ALHE treatments also have both advantages and disadvantages. Regardless of the treatment method, recurrence and incomplete resolution of the disease are frequent. Indeed, a study by Adler et al.^3^ documented that the rate of treatment success was low with excision (40.8%), pulsed dye laser (50.0%), or carbon dioxide laser (54.6%); intermediate for argon laser (66.7%), intralesional corticosteroids (79.1%), or cryotherapy (80.5%); and high for systemic (87.8%) and topical corticosteroids (98.2%), oral pentoxifylline, and isotretinoin (100%). Given the availability of several different treatment modalities, the use of multiple treatments for ALHE is proposed in the dermatologic literature. Selection of appropriate methods should be predicated on parameters such as the number of lesions, the lesion size and site, and previous treatments.

Radiotherapy is currently widely applied for the treatment of tumors, but there are few studies concerning the treatment of ALHE with radiotherapy. Conill et al.^23^ successfully used orthovoltage radiation therapy to treat an ALHE patient who had multiple nodules involving the skin, subcutaneous tissue, and bone of the distal phalanx of the fingers, but this approach carries a risk of impaired function and radiation damage to surrounding normal tissues.

^32^P is a pure β-particle-emitting radionuclide that has a half-life of 14.3 d. The biologic effects of ionizing radiation,^24–26^ benign proliferative diseases,^27^ and malignant tumors can be treated using ^32^P brachytherapy, a form of radiation therapy.^25,26^ In this technique, the radiation source is placed close to the area to be treated,^24–27^ similar to other surface-mold isotope brachytherapies.^28^ The range of electrons emitted by ^32^P is limited in that the maximum range in the tissue is 7.5 mm. The therapeutic range of ^32^P brachytherapy in tissue is about 2 mm, and the accumulated dose falls to subcritical levels within 10 mm of the radioactive source, thus making this approach safe for treatment of lesions in humans^26–27^ and ensuring that surrounding healthy tissue receives only minimal radiation doses. ^32^P brachytherapy may be superior to conventional external beam radiation for treating patients who have small, primary, and/or superficial lesions in terms of providing excellent functional and cosmetic results.^28^ In the case that the authors described in this study, the lesions were located in the preauricular region and its base. The lesions were poorly delineated multiple modules that impeded local surgical removal. ^32^P brachytherapy was performed with improved presentation over 8 years of follow-up. Thus, ^32^P brachytherapy may be a promising new treatment option for ALHE that produces favorable long-term outcomes.

## Conclusions

ALHE is an uncommon, benign, and idiopathic vascular lesion that often has a chronic course with frequent relapses. Many approaches that have both advantages and disadvantages for treating ALHE have been attempted, and there is ongoing debate regarding optimal management for this condition. ^32^P brachytherapy, a form of radiation therapy that places the radiation source close to the area to be treated, could represent a novel method to treat ALHE that produces favorable long-term outcomes. ^32^P brachytherapy may thus be a good choice for recurrent ALHE and for ALHE patients who refused other treatments.
